# Nodule unguéal chez les sportifs: penser à l'exostose sous unguéale

**DOI:** 10.11604/pamj.2015.20.402.6766

**Published:** 2015-04-23

**Authors:** Ouakrim Redouane, Ahmed El Bardouni

**Affiliations:** 1 Service de Chirurgie Orthopédique et Traumatologie, CHU Ibn Sina, Rabat, Maroc

**Keywords:** Exostose, sous unguéale, sportif, nodule, Exostosis, subungual, Athlete, nodule

## Image en medicine

L'exostose sous unguéale est une tumeur ostéo-articulaire bénigne relativement rare, à tendance récidivante, touchant essentiellement le gros orteil. L’étiopathogénie est inconnue, plusieurs théories ont été avancées dont la principale est en rapport avec la notion de traumatisme ou microtraumatisme. Plusieurs diagnostics différentiels doivent être évoquer en particulier le chondrome phalangien, ostéo-chondrome, fibrome sous unguéale, et le mélanome achromique. L'excision chirurgicale reste le principal traitement avec un risque de récidive non négligeable. Nous rapportons le cas d'un homme de 28 ans, sans antécédents particuliers, footballeur professionnel, qui a consulté pour un nodule sous unguéal douloureux du gros orteil droit évoluant depuis 4 mois sans autres signes associés. L'examen clinique a révélé un nodule dur de 15 mm de diamètre, de couleur chair, siégeant au niveau sous unguéal. Le reste de l'examen somatique a été normal. Une radiographie osseuse du pied a révélé une excroissance osseuse en continuité avec la phalangette, à développement latéral. Ce qui a permis de confirmer le diagnostic d'une exostose sous unguéale. Une exérèse chirurgicale a été réalisée. Les suites opératoires ont été simples. Le patient n'a pas fait de récidive avec un recul de 1 an.

**Figure 1:**
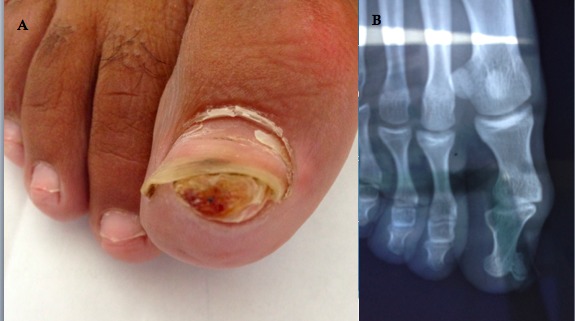
(A) nodule sous unguéal; (B): exostose du gros orteil droit

